# Allele-specific expression variation at different ploidy levels in *Squalius alburnoides*

**DOI:** 10.1038/s41598-019-40210-8

**Published:** 2019-03-06

**Authors:** Isa Matos, Miguel P. Machado, Manfred Schartl, Maria Manuela Coelho

**Affiliations:** 1 Faculdade de Ciências, cE3c- Centro de Ecologia, Evolução e Alterações Ambientais, Departamento de Biologia Animal, Universidade de Lisboa Campo Grande, 1749-016 Lisboa, Portugal; 2 University of Würzburg, Biozentrum, Physiological Chemistry, Am Hubland, Würzburg, Germany; 3 Present Address: Instituto de Microbiologia, Instituto de Medicina Molecular, Faculdade de Medicina, Universidade de Lisboa, Lisbon, Portugal; 4 Comprehensive Cancer Center, University Clinic Würzburg, Josef Schneider Straße 6, 97074 Würzburg, Germany; 5 Hagler Institute for Advanced Study and Department of Biology, Texas A&M University, College Station, USA

## Abstract

Allopolyploid plants are long known to be subject to a homoeolog expression bias of varying degree. The same phenomenon was only much later suspected to occur also in animals based on studies of single selected genes in an allopolyploid vertebrate, the Iberian fish *Squalius alburnoides*. Consequently, this species became a good model for understanding the evolution of gene expression regulation in polyploid vertebrates. Here, we analyzed for the first time genome-wide allele-specific expression data from diploid and triploid hybrids of *S. alburnoides* and compared homoeolog expression profiles of adult livers and of juveniles. Co-expression of alleles from both parental genomic types was observed for the majority of genes, but with marked homoeolog expression bias, suggesting homoeolog specific reshaping of expression level patterns in hybrids. Complete silencing of one allele was also observed irrespective of ploidy level, but not transcriptome wide as previously speculated. Instead, it was found only in a restricted number of genes, particularly ones with functions related to mitochondria and ribosomes. This leads us to hypothesize that allelic silencing may be a way to overcome intergenomic gene expression interaction conflicts, and that homoeolog expression bias may be an important mechanism in the achievement of sustainable genomic interactions, mandatory to the success of allopolyploid systems, as in *S. alburnoides*.

## Introduction

By the classical Mendelian rules of inheritance for traits with intermediate phenotypes an equal contribution from the maternally and paternally inherited alleles to the overall expression was the intuitive solution. Doubts about this equal parental contribution to intermediate phenotypes have been raised, especially in respect to hybrids and polyploids^1–3^. However, still to date, information’s on allele-specific expression (ASE) are scarce and come just from a handful of organisms. To understand the biological meaning of ASE under physiological conditions and in organisms with special genomic situations like hybrids and polyploids, the high-throughput sequencing technologies allow to generate transcriptome-wide data from experimental model systems and non-model organisms.

**Figure 1:**
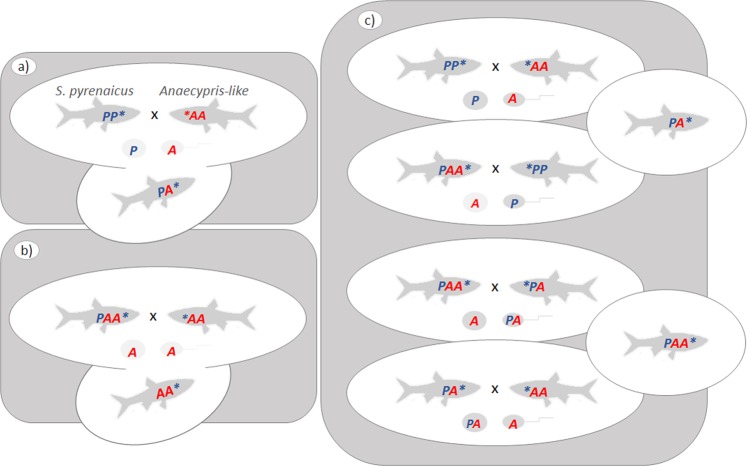
Simplified overview of the *S. alburnoides* reproductive complex. (**a**) Initial interspecific hybridization at the origin of the complex, between females of *Squalius pyrenaicus* (PP genome) and males of an already extinct species belonging to the *Anaecypris hispanica* lineage (AA genome); (**b**) Anaecypris-like nuclear genomotype reconstitution within the complex; (**c**) crosses leading to diploid PA and triploid PAA hybrid *S. alburnoides*. Asterisk represents mitochondrial genotype. This figure covers only the *S. alburnoides* genomotypes involved in this study.

In allopolyploid organisms, two or more sets of diverged genomes are joined through hybridization. Consequently, the gene copies that originated from each parent (homoeologs) may be quite different^4,5^. The differences may be at the level of DNA sequence either in the promoter and/or within the transcribed region of a gene^4,5^. Also, differences can be due to varied chromatin modifications and imprinting^6^. In any case, such differences between homoeologs may result in differential transcription rates and/or differential transcript decay between transcripts derived from each homoeolog, a phenomenon called homoeolog expression bias (HEB)^7,8^. Studies on allopolyploids that have analyzed HEB are numerous for plants and go back several years^9–15^. However, the same phenomenon was only much later described in a vertebrate organism^16,17^, the allopolyploid teleost fish *S. alburnoides* complex. The allopolyploid cyprinid *Squalius alburnoides* is a freshwater fish endemic from the Iberian Peninsula. It resulted from interspecific hybridization between females of *Squalius pyrenaicus* (PP genome) and males of an already extinct species belonging to the *Anaecypris hispanica* lineage (AA genome) (Fig. 1a). The *Anaecypris hispanica*-like lineage now exists only as an all-male line of nuclear non-hybrid diploids (AA nuclear genomotype and *S. pyrenaicus* mitochondrial DNA), reconstituted from hybrids within the complex (Fig. 1b). In the southern Portuguese River basins, the complex consists of fertile forms of different ploidy levels and relative genomic proportions, with PA and PAA genomotypes being the most abundant ones (Fig. 1c), with estimated genome sizes of 2.46 ± 0.044 pg.cell^−1^ and 3.6 ± 0.043 pg.cell^−1^, respectively^18^. *S. alburnoides* combines a set of curious characteristics, such as highly diverse reproductive modes and highly biased sex ratios, reviewed in^19,20^. For example, PAA individuals are preponderantly females, while the nuclear non-hybrid diploids (AA genomotype) are all-males^19,20^.

Previously, based on the analysis of a set of 7 genes, it was shown that a gene-regulatory mechanism involving allelic silencing (AS), which is the most extreme case of HEB, contributes to the regulation of gene expression in allotriploid *S. alburnoides* individuals. The expression patterns were shown to vary according to the gene and organ analyzed, suggesting a considerable plasticity in the process and rejecting the hypothesis of whole haplome silencing^16,17^.

In addition to the phenomenon of AS, also gene expression dosage compensation was described to occur in *S. alburnoides*, reducing the allotriploid expression levels to the same levels of the hybrid diploid counterparts^16,17,21^. Through an indirect approach and with limited number of markers, a dosage effect in a hybridogenetic triploid vertebrate, the frog *Pelophylax esculentus*, had previously been reported^22^. Studies on the *S. alburnoides* complex led to the hypothesis that a causal link exists between AS and the observed dosage compensation^16^. It was suggested that a consistent silencing of one of the three alleles (irrespective which one) across the allotriploid *S. alburnoides* genome could be a reason for the observed similar expression levels between diploid and triploid *S. alburnoides* specimens (a relative expression ratio between diploid and triploid organ samples, consistent with an 1:1 ratio, was the criteriium to assume the same expression level between the two ploidy levels)^16^. To explore that hypotheses, a more detailed knowledge on how expression regulation occurs at the whole genome level in this allopolyploid species was necessary.

Following the initial discoveries of^15,16^, that were based just on a handful of genes, we applied for the first time in a naturally occurring allopolyploid vertebrate a whole transcriptome sequencing approach to the study of homoeolog specific expression (HSE) to help to clarify the role and implications of HEB, and AS, in the complex problem of odd genome regulation and allopolyploid perpetuation. We hoped to clarify if HEB and AS (balanced or unbalanced) are genome wide transcriptomic phenomena or are restricted to subset of genes. Also important is to see if there is a preference towards one of the homoeologs to be down regulated or silenced and if there is a predisposition of certain genes to be affected by HEB. We hope also to clarify if and how does ploidy level increase affect HEB and AS.

In the present work we showed that HEB is quite extensive, but the full silencing of one of the alleles, which is the most extreme HEB scenario, is seen only for a minority of genes. Moreover, AS is not a genomewide transcriptomic phenomenon that systematically silences one of the alleles in *S. alburnoides* triploids, as previously thought.

## Results

### Genome specific expression patterns in livers of diploid and triploid hybrids

**Figure 2:**
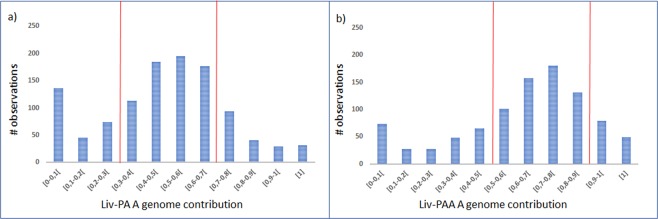
Distribution of transcripts according to A genome contribution to the overall gene specific transcription in liver samples. Distribution in (**a**) liv-PA library and (**b**) liv-PAA library. Vertical red lines represent the considered boundaries for balanced allelic expression.

We first considered single-nucleotide variants (SNV’s) for HSE quantification in liver tissue and used 2807 single-nucleotide polymorphisms (SNPs) in liv-PA, distributed over 1121 transcripts, and 2305 SNPs in liv-PAA distributed over 937 transcripts. Plotting the distribution of transcripts according to the fraction of A genome contribution reveals the pattern of genome specific expression in diploid (Fig. 2a) and triploid (Fig. 2b) hybrid liver. Transcripts that exhibit a fraction larger than 0,5 are those that mapped more A than P reads in the hybrids. Those with ratios of less than 0,5 are transcripts from genes where the P alleles were expressed at a higher level than the A alleles. Following the criteria of ^23^, we assume as balanced allelic expression profile for diploids, genes with less than 70% of expression preference of one the alleles (Fig. 2a). We found for liv-PA 764 of 1121 transcripts (68%) with balanced homoeolog expression and 357 transcripts (32%) showing strong homoeolog expression bias (Table 1). Additionally, we observed a significant unbalanced homoeolog expression bias (χ^2^, p < 0,05) towards P-genome alleles, meaning that for a significant number of genes displaying homoeolog expression bias, the P allele is preferentially expressed.

**Table 1:** Genome specific expression fractions in livers of diploid and triploid *S. alburnoides* hybrids.

	BHE	HEB	HEB (P)	HEB (A)	MGE (P)	MGE (A)	Total
Liv-PA	764 (68%)	357 (32%)	255 (23%)	102 (9%)	136 (12%)	32 (3%)	1121
Liv-PAA	649 (69%)	289 (31%)	240 (26%)	49 (5%)	73 (8%)	49 (5%)	938

BHE- Balanced homoeolog expression; HEB-Homoeolog expression bias; HEB(P)-Homoeolog expression bias towards P genome; HEB(A)-Homoeolog expression bias towards A genome; MG(P)-Monogenomic expression of P alleles; MG(A)-Monogenomic expression of A alleles.

We then extrapolated the criteria used in^23^ to triploids and considered that for a ratio of two A alleles to one P allele, balanced expression between both genomotype alleles would result in an A genome expression fraction between 0,5 and 0,9 (Fig. 2b). For liv-PAA we found 649 of 938 transcripts (69%) with balanced homoeolog expression and 289 transcripts (31%) showing strong homoeolog expression bias (Table 1). Focusing on the transcripts presenting strong homoeolog expression bias, it was observed that as for the diploid form, the homoeolog expression bias is shifted significantly (χ^2^, P < 0,05) towards higher expression of the P alleles (Table 1).

From the transcripts that present strong allelic bias, we considered under allelic silencing the ones exhibiting a fraction of allelic contribution lower than 0,1. From the total analyzed genes 15% in Liv-PA and 13% in Liv-PAA follow under this category (Table 1). Interestingly, we found that the number of transcripts presenting HEB is not significantly affected (χ^2− test^, P > 0,05) by ploidy level, with both diploid and triploid showing similar number of genes with HEB, but also AS.

### Genome specific contribution in hybrid diploid and triploid juveniles

**Figure 3:**
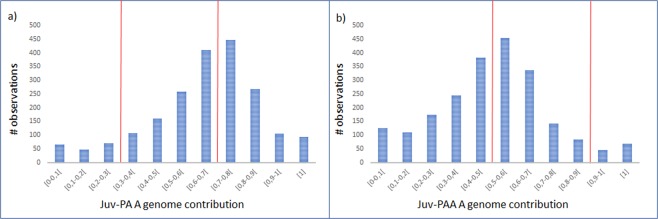
Distribution of transcripts according to A genome contribution to the overall gene specific transcription in juvenile samples. Distribution in (**a**) juv-PA library and (**b**) juv-PAA library. Vertical red lines represent the boundaries considered for balanced allelic expression.

The effective number of SNVs considered for the HSE quantification was 5424 SNPs in juv-PA, distributed over 2039 transcripts and 5840 SNPs in juv-PAA distributed through 2169 transcripts. Again, we plotted the distribution of transcripts according to the fraction of A genome contribution, revealing the pattern of genome specific expression in diploid (Fig. 3a) and triploid hybrid juveniles (Fig. 3b). Using the same criteria as for the liver expressions, we found for juv-PA 1386 out of 2039 transcripts (68%) presenting balanced homoeolog expression and 653 transcripts (32%) showing strong homoeolog expression bias (Table 2). A significant unbalanced homoeolog expression bias (χ2-test, p < 0,05) was also observed but different from liver. In Juv-PA the bias was towards A, meaning that from the pool of transcripts displaying HEB, for a significant majority, A alleles were higher expressed.

**Table 2:** Genome specific expression fractions in juveniles of diploid and triploid *S. alburnoides* hybrids.

	BHE	HEB	HEB (P)	HEB (A)	MGE (P)	MGE (A)	Total
Juv-PA	1386 (68%)	653 (32%)	184 (9%)	469 (23%)	66 (3%)	94 (5%)	2039
Juv-PAA	1064 (49%)	1105 (51%)	1038 (48%)	67 (3%)	125 (6%)	67 (3%)	2169

BHE- Balanced homoeolog expression; HEB-Homoeolog expression bias; HEB(P)-Homoeolog expression bias towards P genome; HEB(A)-Homoeolog expression bias towards A genome; MGE(P)-Monogenomic expression of P alleles; MGE(A)-Monogenomic expression of A alleles.

In juv-PAA we found 1064 SNPs in 2169 transcripts (49%) presenting balanced allelic expression and 1105 genes (51%) showing strong allele expression bias (Table 2). Significant unbalanced homoeolog expression bias (χ^2-test^; p < 0,05) was observed towards P (Table 2). From the total analyzed genes, 8% in Juv-PA and 9% in Juv-PAA present (either A or P) monogenomic expression (Table 2).

In the case of the juvenile dataset, we found that, unlike in adult liver, the number of transcripts presenting HEB is significantly affected (χ^2- test^, P < 0,05) by ploidy level.

### Functional enrichment analysis

We performed a Gene ontology (GO) and a Kyoto Encyclopedia of Genes and Genomes (KEGG) pathway enrichment analysis in each of the defined monogenomic expression (MGE) groups of transcripts. We found significant functional enrichment of several terms (Tables 3 and 4). In essence, genes that underwent preferential silencing of the A alleles were enriched in ribosomal-linked terms (in juvenile samples, but also in both ploidy levels irrespective of the tissue type) while genes preferentially silenced for the P allele showed an enrichment of mitochondrial function related terms (in liver samples and both ploidy levels).

**Table 3:** Functional enrichment in gene ontology (GO) terms and KEGG pathways of A and P monogenomic expressing (MGE) gene groups in liver and juveniles’ libraries irrespective of ploidy level.

	MGE	Category	Term description	#	p
*Liver*	**P**	CC	Intratracellular part	28	1,10E-02
		CC	cytoplasm	21	1,90E-02
		CC	intracellular	28	3,10E-02
	**A**	NS			
*Juveniles*	**P**	NS			
	**A**	BP	translation	10	8,60E-04
		BP	peptide biosynthetic process	10	4,80E-04
		BP	organonitrogen compound biosynthetic process	12	5,90E-04
		BP	amide biosynthetic process	10	5,00E-04
		BP	peptide metabolic process	10	6,50E-04
		BP	organonitrogen compound metabolic process	13	1,30E-03
		BP	cellular amide metabolic process	10	1,60E-03
		BP	cellular protein metabolic process	17	1,50E-02
		BP	protein metabolic process	18	2,30E-02
		CC	cytosolic part	8	6,40E-06
		CC	cytosolic ribosome	6	8,30E-05
		CC	ribosome	7	9,40E-05
		CC	cytosolic large ribosomal subunit	5	1,30E-04
		CC	ribonucleoprotein complex	9	2,10E-04
		CC	intracellular ribonucleoprotein complex	9	2,10E-04
		CC	ribosomal subunit	6	1,90E-04
		CC	large ribosomal subunit	5	3,50E-04
		CC	intracellular part	30	3,80E-04
		CC	cytoplasm	23	7,80E-04
		CC	cytoplasmic part	18	8,00E-04
		CC	cytosol	8	2,50E-03
		CC	intracellular	30	2,70E-03
		CC	intracellular non-membrane-bounded organelle	12	4,50E-03
		CC	non-membrane-bounded organelle	12	4,50E-03
		CC	macromolecular complex	16	7,10E-03
		CC	intracellular organelle	23	2,80E-02
		CC	organelle	23	3,20E-02
		MF	structural constituent of ribosome	8	3,70E-05
		MF	structural molecule activity	9	7,20E-04
		MF	rRNA binding	4	4,00E-03

(BP) Biological process, (MF) molecular function, (CC) cellular component, (NS) no significantly enrichment, (#) number of transcripts, (p) Benjamini corrected p-value. GO enrichment analysis was performed considering all levels of classification of terms.

**Table 4:** Functional enrichment in gene ontology (GO) terms and KEGG pathways of A and P monogenomic expressing (MGE) gene groups in diploid (PA) and triploid (PAA) libraries irrespective of the source tissue type.

	MGE		Term description	#	p
*PA*	**P**	BP	ATP biosynthetic process	4	8,00E-02
		CC	respiratory chain	4	3,90E-02
	**A**	BP	peptide biosynthetic process	3	4,50E-02
		BP	peptide metabolic process	3	3,80E-02
		BP	cellular amide metabolic process	3	4,10E-02
		MF	structural molecule activity	4	1,90E-03
		MF	structural constituent of ribosome	3	7,40E-03
*PAA*	**P**	BP	carbohydrate derivative biosynthetic process	8	4,50E-02
	**A**	BP	translation	5	1,00E-02
		BP	peptide biosynthetic process	5	5,50E-03
		BP	amide biosynthetic process	5	5,20E-03
		BP	peptide metabolic process	5	4,90E-03
		BP	cellular amide metabolic process	5	7,20E-03
		BP	organonitrogen compound biosynthetic process	5	1,60E-02
		MF	structural constituent of ribosome	4	2,60E-03
		MF	structural molecule activity	4	1,80E-02
		KE	Ribosome	3	2,80E-02

(BP) Biological process, (MF) molecular function, (CC) cellular component, (KE) KEGG pathway, (NS) no significant enrichment, (#) number of transcripts, (p) Benjamini corrected p-value. GO enrichment analysis was performed considering all levels of classification of terms.

We further analysed the genes affected by MGE and identified transcripts with consistent P or A monogenomic transcriptional contribution in all four libraries (Table 5). Within our criteria of significance, no functional enrichment was detected in either of these two groups. However, if considering the gene function of each of these above mentioned MGE genes, the link of allelic silencing to mitochondria and to ribosomes is also seen.

**Table 5:** Transcripts with consistent P and consistent A monogenomic transcriptional contribution regardless of sample type and ploidy level.

MGE	Unigene ID	Ref. Gene ID	Ref. Sequence	Symbol	Definition
P	Unigene101062	gi|318054652	NP_001187754.1	NDUFC2	NADH dehydrogenase (ubiquinone) 1 subunit c2 [Ictalurus punctatus]
	Unigene114185	gi|41053742	NP_957180.1	GCDH	Glutaryl-CoA dehydrogenase a [Danio rerio].
	Unigene2212	NaN			
	Unigene43419	gi|47087309	NP_998647.1	ABCD3	ATP-binding cassette sub-family D member 3 [Danio rerio]
	Unigene45629	gi|41055873	NP_957287.1		Uncharacterized protein LOC393968 [Danio rerio]
	Unigene48654l	gi|47550715	NP_999871.1	HNRNPA0	Heterogeneous nuclear ribonucleoprotein A0b [Danio rerio]
A	Unigene100595	gi|18859307	NP_571384.1	Ran	GTP-binding nuclear protein Ran [Danio rerio]
	Unigene101028	gi|18858719	NP_571660.1	FTH1	Ferritin heavy chain [Danio rerio]
	Unigene114523	gi|47523975	NP_998887.1	SLC25A3	Solute carrier family 25 member 3 [Danio rerio]
	Unigene122169	gi|225715740	gb|ACO13716.1	TIMM8A	Mitochondrial import inner membrane translocase subunit Tim8 A [Esox lucius]
	Unigene134826	gi|51010975	NP_001003447.1	RPL15	60S ribosomal protein L15 [Danio rerio]
	Unigene140252	gi|124300811	dbj|BAF45901.1	RPS13	Ribosomal protein S13 [Solea senegalensis]
	Unigene146647	gi|55250139	gb|AAH85596.1		Zgc:153867 protein [Danio rerio]
	Unigene23269	gi|47086529	NP_997925.1	RPL17	60S ribosomal protein L17 [Danio rerio]

MGE- monogenomic expression; UniGene ID from the *de novo* transcriptome assembly of *S. alburnoides* complex; Ref. Gene ID- Gene ID.

## Discussion

In this work we describe homoeolog expression in the *S. alburnoides* complex, comparing the profiles of natural occurring diploid with triploid individuals to better understand the mechanisms of odd genome regulation and perpetuation in a successful allopolyploid vertebrate. It is the first transcriptomic attempt to quantify ASE in a natural allopolyploid fish.

Despite the PCR based approaches undertaken previously^16,17^ proved to be sensitive and valuable assays for an initial assessment of the gene expression profile in the *S. alburnoides* complex, technically they were constraint to the analysis of a few genes with a known sequence.

Next-generation sequencing technologies brings together the advantages of high-throughput and high-sensitivity to the study of gene expression. The RNA-Seq approach allowed to look at the gene expression in the *S. alburnoides* complex at a much broader and integrative range. It allowed us to distinguish the different genome-specific gene copies and how they contribute to the overall expression of each gene for a much higher number of genes, and to draft a comparative profile of allele specific expression between diploid and triploid S*. alburnoides* fish.

When analyzing the genome-specific expression contribution per gene in the *S. alburnoides* complex, we found biased contribution of homoeologs that ranged from subtle differences to complete silencing of alleles, irrespective of the ploidy level or sample type. However, when comparing the results obtained for liver and for juveniles, results concordance is scarcer. We hypothesize that the lack of concordance between the liver and juveniles’ datasets is mostly due to the intrinsic difference between a single organ and a whole animal. The impact of such differences on the output of gene expression profiling is well-documented^8,24–28^. A feature not yet explored is the intrinsic age-dependent hepatic polyploidization process. At least in mammals it is a feature of normal adult livers to progressively increase the amount of polynucleated hepatocytes, with humans presenting up to 50% polyploid cells and mouse up to 90%^29^. However, for non-mammals, and specifically in fish, we are not aware of an extensive existence of this phenomenon, but it is a concept worth to be considered. Nevertheless, comparing homoeolog expression for such different sample types as liver and juvenile full body samples seems to be not very informative.

When analyzing the genome-specific expression contribution per gene in *S. alburnoides*, we found extreme biased contribution of homoeologs (as defined by^23^) in more than 30% of the considered transcripts, irrespective of the ploidy level or sample type. Hence, a considerable fraction of the genome is strongly affected by HEB. This result may even be an underestimation. As discussed by^30^, the study of allele specific expression in full body samples and even in single tissue or organ samples can give skewed and/or diluted signals. The results we obtained imply that equally balanced allelic expression is not a necessary regulatory condition to achieve appropriate amounts of gene product in fish of the *S. alburnoides* complex, neither as relevant factor to explain the success of allopolyploid *S. alburnoides* nor as distinctive mechanism between diploid and triploid *S. alburnoides* biotypes.

Gene expression is governed at the level of transcription by interactions between cis- and trans-acting regulatory elements^31,32^. When in hybrids unequal expression of parental alleles has been observed, it has been considered as a signature of cis-regulatory divergence^31^. This may also apply for the significant degree of HEB which was found in the intergeneric hybrids of *S. alburnoides*. Evidence is accumulating that also in non-hybrid genomes, the variation found in regulatory regions between alleles is sufficient to affect the level of expression of the two variants^4,27^. For example, significant cis regulatory variation in 80% of mouse genes have been found^33^ and allelic imbalance was estimated to affect greater than 89% of genes of mouse, cow and humans, in at least one tissue^27^.

A different picture emerges from a study of another hybrid fish, the Amazon molly (*P. formosa*). Here, allele specific gene expression analysis from different organs, including liver, brain and ovary revealed only a very small percentage of genes (between 1.2 and 4.1%) presenting HEB^34^. However, *P. formosa* is a clonal hybrid organism, resultant from a *single time* successful hybridization event at least 100.000 years old^34,35^. This makes *P. formosa* much different from the reproductive complex of *S. alburnoides*, which results from a continuum of intricated networks of genetic exchanges, *de novo* hybridizations and ploidy levels shifts^36^. The old “frozen” hybrid genomic context of *P. formosa* may have evolved mechanism that counteract HEB.

An important feature of the homoeolog expression profile is whether this expression is balanced or unbalanced^7^. Balanced homoeolog expression means that expression does not favor one component genome, while in unbalanced homoeolog expression one of the intervenient genomes is favored^8^. We found a significant unbalanced homoeolog expression in all *S. alburnoides* libraries. In all liver samples there was consistency in terms of magnitude and bias direction towards the P genome. Conversely, in the whole-body juvenile samples, the ploidy state appears to influence the direction of the bias. HEB was skewed towards A in Juv-PA sample while it is displaced towards P in juv-PAA. In liver, ploidy level did not significantly affect neither the extent of HEB nor the tendency towards preferential expression of the P alleles. Those results imply that balanced allelic expression is not a regulatory necessity to cope with elevated ploidy in *S. alburnoides*. Our data also supports the notion that homoeolog specific expression in diploid and triploid *S. alburnoides* liver is not a simple additive phenomenon. It was previously shown^21^ that the quantitative expression profiles of livers from the *S. alburnoides* parental genomotypes (AA and PP) are significantly different. Most transcripts were found at much higher levels in AA than in PP livers^21^. Taken this into consideration, a simplistic model of additive homoeolog expression in allopolyploid *S. alburnoides* could be only put forward if we had found homoeolog expression bias towards A homoeologs, but not towards P, as it was the case. As many interactions between divergent regulatory machineries occur, new patterns of gene expression and homoeolog regulation may be more complex and difficult to predict. In plants, reshaping of homoeolog expression has been commonly found^5,31,37^. It was also noted in this context that alterations in to the original expression pattern of the originally non-dominant genome occurred^37^.

Our results from *S. alburnoides*, are in line with studies from other systems, where unbalanced expression has been commonly observed in plant hybrids of different ploidies^11,12,38–42^. Notably, in cotton, significant differences between studies, in terms of the magnitude of the expression bias and bias direction, have been found^37,43,44^.

While we observed for liver samples that the extent of HEB was not significantly affected by ploidy level (2n vs 3n), in juveniles there was a significantly higher number of transcripts in 3n than in the 2n juveniles presenting HEB.

Unequal expression of parental alleles has been pointed out in diploid hybrid plants as a signature of cis-regulatory divergence, because both parental alleles should be proportionally exposed to the same set of trans-acting regulators^31^. However, for an unorthoploid (increased and uneven ploidy level hybrid) this assumption is not straightforward since the network of interactions between cis and trans regulators is unpredictably influenced by the unbalanced contribution of parental genomes and increased number of non-additive interactions between the parental genomes^5^.

The extent of HEB ranged in our analysis from only subtle allelic differences to complete silencing of one (or more) alleles. Thus, we considered another phenomenon, allele-specific silencing or monogenomic gene expression, where expression is derived from only one of the parental genomes. In diploid hybrids, when transcription from only one allele was detected it is undoubtful to infer that the other allele is silenced. In the case of allotriploid *S. alburnoides* of PAA genomic composition, when only P genome derived expression was detected at any locus this has to result from silencing of both A homoeologs at that locus. However, in the case of expression only from the A genome we cannot conclude if A transcripts are coming from one or from both A alleles. Thus, exclusive expression of A can mean either biallelic or monoallelic expression.

In diploid *S. alburnoides* (PA), even though most transcripts analyzed presented biallelic expression, we detected for the first time in this fish model the occurrence of MGE, either from the A or P homoeologs. This is in accordance with several studies on other diploid organisms, where transcription from only one allele has been found not only due to sex-chromosome inactivation and genomic imprinting but also stochastic silencing in autosomal genes (reviewed in^30^).

In triploid *S. alburnoides* (liv-PAA and juv-PAA) we found, besides the biallelic expression, monoallelic expression of P but also P allelic silencing. P homoeolog silencing in triploid *S. alburnoides* has already been reported previously^16,17,21,36^, but monoallelic P expression has not been observed so far in *Squalius* genus. This new finding of expression from only one allele in the context of allotriploidy in this fish, agrees with a previous report of the same pattern in another successful allopolyploid complex, the allopolyploid *Poecilia formosa*^36^.

To explain the first observations of P allele specific silencing in triploid *S. alburnoides*, based on the analysis of a limited number of genes^16^, a parsimonious hypothesis was suggested postulating that one of the three alleles, irrespective which one, could be systematically silenced across the entire *S. alburnoides* genome. This was proposed as explanation why triploids presented similar expression levels to their diploid counterparts for the set of analyzed genes. However, our genome wide analyses show that allelic silencing does not happen genome-wide in triploid *S. alburnoides*, and additionally shows that AS can also be found in diploid S. alburnoides, and at the same extension than in the triploids.

Despite heterosis and hybrid vigor are well known phenomena associated with hybrids^45^, not all crosses result in heterosis and some hybrids do not even survive and/or reproduce^46,47^. Traits derived from different genetic backgrounds merged in the hybrids may not be fully compatible, and fitness can be reduced. A possible explanation for the success of some hybrids like *S. alburnoides* may come from gene expression plasticity^21^, where ASE regulation at each locus may have a significant role.

We investigated also the biological context of the AS occurrence in *S. alburnoides*. As mentioned, upon hybridization (and ploidy increase) disruption of well-established interlocus interactions may reveal incompatibilities^46,47^, so many hybrids and allopolyploids may either be non-viable or suffer from reduced fitness. Mito-nuclear incompatibilities have been found to influence hybrid inviability^48,49^. More specifically, there is evidence of hybrid incompatibilities between nuclear-and mitochondrial DNA (mtDNA)-encoded elements^50^, for example the interaction between nuclear- and mtDNA-encoded subunits of the oxidative phosphorylation (OXPHOS) proteins^51,52^.

In the *S. alburnoides* complex, apart from a few exceptions^53,54^, there is almost exclusive presence of *S. pyrenaicus* (P) mtDNA^19^. In that sense, it is interesting to note that P monogenomic expression was associated to mitochondria related GO terms, irrespective of ploidy level and sample type. We thus hypothesise that expression of only the P alleles of mitochondria related loci in *S. alburnoides* specimens might be an effective way to cope with incompatibilities of the hybrid genome and the P derive mitochondria^50^. For instance, by facilitating or optimizing mitochondrial-nuclear interactions through reducing post-transcriptional and translational incompatibilities between the PA(A) nuclear DNA and the maternally inherited P-only mtDNA. This is in accordance with the mitonuclear coadaptation theory^55^, which postulates that nuclear genes that interact with mitochondria are expected to be maternally biased. Also, we found a tendency towards monogenomic A transcriptional activity of genes related to ribosomes. Assembly of ribosomes involves more than 300 proteins and RNAs^56^. The genes that code for RNA molecules constitute the ribosomal sub-units and are organized in tandem repeats at chromosomal regions called Nucleolar Organizing Regions (NORs)^57^. But, not all NORs are transcriptionally active. It was previously found that *S. pyrenaicus* presented only one pair of chromosomes with active NORs, while all forms of *S. alburnoides* presented mostly multichromosomal active NORs. Hence, the observed increased NOR numbers in the *S. alburnoides* complex specimens would be A genome derived^58^. Accordingly, it can be assumed that at a given time, there is a high probability to find more A-genome derived than P-genome derived ribosomal RNA molecules, as both PA and PAA genomotype individuals would have only one P-derived competent NOR per cell while having multiple A-derived NORs. Also, in accordance with the gene balance hypothesis^59,60^ which posits that in multi-subunit complexes, changes in the stoichiometry of the components of those complexes is deleterious, an intergenomic ribosomal gene conflict can be speculated to support the tendency we found towards monogenomic A transcriptional activity of ribosomal related genes.

Worthy of additional considerations is the fact that cellular ploidy increase is accompanied by a proportional (but not linear) increase in cell size. However, this increase is not significantly reflected at the full body level in many animal polyploids, including *S. alburnoides*.

Knowing that one of the key roles of mitochondria is the metabolic regulation of cell size, by setting the growth rate through metabolic activity and affecting the balance between growth and proliferation^61^, assuring mitonuclear compatibilities, possibly through HEB and AS, may be required to reach an optimal allopolyploid fitness. Work from several authors has shown that even minor inefficiencies in the function of mitochondria can have major fitness consequences for an organism^62–65^. Inefficiencies may lead to inadequate energetic efficiency and undesirable oxidative stress levels^66,67^.

Also, gene expression demands are tuned to cellular volume. Larger cells need to produce and maintain more cellular components (proteins) than do smaller cells^68^. Hence, the need for ribosomes (as of rRNAs and some mRNAs) scales with cell size. Avoidance of an intergenomic ribosomal gene conflict, possibly through HEB and AS, may contribute to efficient translation and optimized cell function and organism homeostasis of the allopolyploid.

Functional studies are required to substantiate these considerations: In conclusion, our results imply that balanced allelic expression is not a necessary regulatory condition to achieve appropriate amounts of gene products in the *S. alburnoides* complex and support that homoeolog specific expression in diploid and triploid *S. alburnoides* is not a simple additive phenomenon.

Despite HEB is quite extensive, the full silencing of one of the alleles as the extreme, was seen only in a minority of genes. However, AS was found mostly in genes related to mitochondria and ribosomes, what lead us to hypothesize that AS may be a way to overcome intergenomic gene expression interaction conflicts. In that sense, HEB and AS may be key players into the achievement of sustainable genomic interactions, mandatory to the success of allopolyploid systems, as the *S. alburnoides* complex.

## Materials and Methods

### Model system

We used the allopolyploid hybridogenetic complex *S. alburnoides* as experimental model to study specific allelic contribution to the transcript pool. “Hybridogenetic” refers to an alternative mode of reproduction and “complex” is the terminus denoting a natural system composed of parental species and their hybrids of different ploidies, with altered modes of reproduction and reproductive interdependence. The *S. alburnoides* complex resulted from a cross of a *Squalius pyrenaicus* female (contributing with the P genome) and an *Anaecypris-like* male (contributing with the A genome) (see^19^ and^20^ for extensive review).

### Libraries

Previously constructed and sequenced RNA-Seq libraries, enriched for mRNA by hybridization with Oligo-dT beeds, have been used in this study (Supplementary Table S1 – all additional files at https://figshare.com/s/c03974866dbd92b5a24d).

In summary:

- From juvenile samples: Three barcoded RNA libraries had been previously constructed and paired-end sequenced using Illumina HiSeq 2000, producing 12 Gb clean data in 3 data sets (juv-AA; juv-PA; and juv-PAA, ~4 Gb per library) of paired-end sequence reads (around 91 bp)^21^.
- From adult liver samples: Four barcoded RNA libraries had been previously constructed, one for *S. pyrenaicus* (liver- PP) and three for *S. alburnoides* (liver- AA, liver-PA and liver-PAA)^21^. The four libraries were paired-end sequenced using Illumina HiSeq 2000, producing 4 Gb of clean data in 4 data sets (Liv-AA; Liv-PA; Liv-PP and Liv-PAA, ~1 Gb per library) of short paired-end sequence reads (around 50 bp)^21^.
- From adult brain and gonad samples: Six barcoded RNA libraries were constructed, two for *S. pyrenaicus* brain and two for gonads^69^, one for *S. alburnoides* nuclear non-hybrid AA male brain and one for its gonad (this study). For the construction of the six libraries, total RNA was purified from individual gonads and brains of two *S. pyrenaicus* (PP) individuals, and from a pool of brains and gonads (separated by tissue type) of a AA *S. alburnoides* and a rare occurring AAA *S. alburnoides*. All libraries were paired-end sequenced using Illumina HiSeq 2000, producing in total 12 Gb of clean data in six data sets (brainF-PP, brainM-PP, gonF-PP, gonM-PP; brain-AA and gon-AA, ~2 Gb per library) of paired-end sequence reads (around 91 bp).

The RNA-Seq fastq files are available through public repositories (Supplementary Table S1 - Additional Files at https://figshare.com/s/c03974866dbd92b5a24d).

### *S. alburnoides* “*de novo*” transcriptome assembly

We used SOAPdenovo to produce a transcriptome *de novo* assembly using all available libraries from individuals participating in the *S. alburnoides* complex (juv-AA, juv-PA, juv-PAA, liv-PP, liv-AA, liv-PA, liv-PAA, brainF-PP, brainM-PP, gonF-PP, gonM-PP, brain-AA and gon-AA) to produce a more comprehensive “*S. alburnoides* breeding complex” reference transcriptome than the one previously available at^21^. Statistics of assembly quality for *S. alburnoides* complex transcriptome provided as Supplementary Table S2 (all additional files at https://figshare.com/s/c03974866dbd92b5a24d). Assemblies were taken into further processes of sequence splicing and redundancy removing with the sequence clustering software TGICL^70^. After clustering, contigs were annotated with blastx and blastn against the NCBI non-redundant protein database (NR) (e-value < 0.00001), retrieving proteins with the highest sequence similarity to the given contigs. We used Blast2GO program^71^ to get functional annotations. Assembly and gene ontology (GO) annotations are available as Datasets in Additional Files at https://figshare.com/s/c03974866dbd92b5a24d.

Using the *de novo* assembled *S. alburnoides* complex transcriptome as reference, we detected and quantified single nucleotide variants (SNVs) between all different samples with SOAPsnp^72^. SNV calling criteria was as following: consensus quality ≥ 20; depth of coverage of the site and the flanking sequences ≥ 3; distance from the last candidate SNV ≥ 5 bp; distance from the borders > 5 bp. SNV calling, and alleles quantification are provided as Supplementary Information.

### Comparative genome specific expression quantification

Several polymorphic sites were detected within each nuclear non-hybrid sample (both *S. pyrenaicus* and *S. alburnoides* AA samples) and binned for separation from the non-polymorphic sites. After excluding the intragenomic polymorphisms, P and A genome specific variants were identified. Only when a single nucleotide variant was detected within all 4 AA genome libraries, and it was different from the single variant found in all 5 PP genome libraries, such site was considered an SNP for the allele specific expression quantification in the hybrid *S. alburnoides* individuals. In our reference transcriptome sequences, which represents a hybrid species, the polymorphic positions are represented by the most frequent or more represented nucleotide base from the pool of all reads covering that site. Thus, as observed by^23^, reads of the same SNV as reference, map with higher efficiency than others, an effect known as reference bias. When inspecting our data, it was obvious that there was a clear tendency towards higher read counts of the reference variant. To validate and calculate this read count bias, we used the intra-genomic polymorphisms. To do so, we started with the assumption of a 1:1 allelic contribution in the complex nuclear non-hybrid forms (*S. pyrenaicus* and *S. alburnoides* AA samples) and calculated the deviation from the expectation (allele1 reads/allele2 reads = 1) at each position. Mean deviation was calculated for each nuclear non-hybrid sample, and a mean value of these means was used as correction factor of read counts of each transcript of the hybrid libraries. brain-AA and gon-AA were excluded from this analysis because of the inclusion of triploid AAA in the AA sample pools.

In the hybrid samples, only nucleotide positions showing 20 or more SNV supporting reads were considered for quantification, increasing the confidence of the quantitative allele/genome-specific expression analysis, but obviously reducing the number of SNV positions to analyze. For the majority of transcripts identified as having SNVs between P and A genomes, more than one site per transcript was identified. In these cases, for each hybrid dataset a mean number of reads per allele/genome and per transcript was calculated.

The number of reads for each variant of the polymorphic site per transcript in the hybrid libraries (PA and PAA) was used to quantify the genome specific and/or allele specific contribution for the overall expression.

A conservative approach was followed to establish the boundaries for balanced expression and the cutoffs to classify HEB and MGE. For diploids we followed the criteria of ^23^, which assume as having balanced allelic expression only genes with less than 70% of expression preference of one of the alleles. For triploids, the same criteria of ^23^ were extrapolated proportionally. We considered that for a ratio of two A alleles to one P allele balanced expression should correspond to an A genome expression fraction between 0,5 and 0,9.

### Functional term enrichment analysis

To infer a possible biological context of genes exhibiting preferential AS in *S. alburnoides*, transcripts in which expression was determined to be coming only from one genome type (P or A) were organized in groups according to genome-specific silencing within tissue/sample type (liver or juveniles) and also according to ploidy level (2n-PA and 3n-PAA). 8 groups of monogenomic derived transcripts were assembled as follows: within liver libraries (independently of ploidy level), group i) of transcripts presenting P monogenomic expression and group ii) presenting A monogenomic expression; within juveniles libraries (independently of ploidy level), group iii) of transcripts presenting P monogenomic expression and group iv) presenting A monogenomic expression; within the PA libraries (independently of sample type), group v) of transcripts presenting P monogenomic expression and group vi) presenting A monogenomic expression; within the PAA libraries (independently of sample type), group vii) of transcripts presenting P monogenomic expression and the group viii) presenting A monogenomic expression. The list of transcripts organized according to genome-specific silencing (from i to viii) is available as Supporting Information in Additional Files (https://figshare.com/s/c03974866dbd92b5a24d).

To perform functional enrichment analysis in each of these groups, we used DAVID Bioinformatics Resource v6.7 (http://david.abcc.ncifcrf.gov/), with default parameters. The top blastx hits in nr database corresponding to each *S. alburnoides* contigs were used as customized reference background and compared to the above-mentioned input lists of monogenomic transcribed genes.

Enriched terms were ranked in the ontology categories Biological Process (BP), Cellular Component (CC) and Molecular Function (MF) and KEGG pathways.

Significant enrichment was only considered when Benjamini corrected p-value was ≤0.05.

## Supplementary information

Supplementary Information

## Data Availability

All additional files and datasets supporting this article are available through the figshare repository. DOI:10.6084/m9.figshare.6825497. (https://figshare.com/s/c03974866dbd92b5a24d).
