# Supplementary material for: Allele-specific expression variation at different ploidy levels in Squalius alburnoides
Source: Sci Rep. 2019 Mar 6;9:3688. doi: 10.1038/s41598-019-40210-8 (PMC6403402; doi:10.1038/s41598-019-40210-8)
Supplement: Supplementary file 1 — Supplementary Information [file 41598_2019_40210_MOESM1_ESM.docx]

Allele-specific expression variation at different ploidy levels in *Squalius alburnoides*

Isa Matos ^1,2^, Miguel P. Machado^1,2,3^, Manfred Schartl*^2,4,5^, Manuela Coelho^1^

^1^, Faculdade de Ciências, cE3c- Centro de Ecologia, Evolução e Alterações Ambientais, Departamento de Biologia Animal, Universidade de Lisboa Campo Grande 1749-016 Lisboa, Portugal.

^2^University of Würzburg, Biozentrum, Physiological Chemistry, Am Hubland, Würzburg, Germany.

^3^ Present Adress: Instituto de Microbiologia, Instituto de Medicina Molecular, Faculdade de Medicina, Universidade de Lisboa, Lisbon, Portugal.

^4^Comprehensive Cancer Center, University Clinic Würzburg, Josef Schneider Straße 6, 97074 Würzburg, Germany.

^5^ Hagler Institute for Advanced Study and Department of Biology, Texas A&M University, College Station, USA

*Correspondence to phch1@biozentrum.uni-wuerzburg.de

**Supplementary Information and Data availability:**

All additional files and datasets supporting this article are available through the figshare repository. DOI: 10.6084/m9.figshare.6825497

(<https://figshare.com/s/c03974866dbd92b5a24d>).

Datasets: de novo assembled transcriptome sequences; functional annotation of transcriptome sequences; SNP calling for all libraries.

Supporting Information: Artificial grouping of transcripts organized according to genome-specific silencing (from i to viii).

Appendices: Table S1 and Table S2.

Table S1: Previously constructed and sequenced libraries that have been used in this study.

| **Libraries** | **Gender** | **Sequencing information** | **Repository** | **Accession** |
| --- | --- | --- | --- | --- |
| ***liv-AA*** | ***male*** | *Matos et al., 2015* | ArrayExpress | E-MTAB-3174 |
| ***liv-PP*** | ***male*** |  |  |  |
| ***liv-PA*** | ***female*** |  |  |  |
| ***liv-PAA*** | ***female*** |  |  |  |
| ***juv-AA*** | ***nd**** | Matos et al., 2015 | ArrayExpress | E-MTAB-3174 |
| ***juv-PA*** | ***nd*** |  |  |  |
| ***juv-PAA*** | ***nd***** |  |  |  |
| ***gonF-PP*** | ***female*** | *Machado et al., 2016* | ENA | PRJEB9465 |
| ***gonM-PP*** | ***male*** |  |  |  |
| ***brainF-PP*** | ***female*** |  |  |  |
| ***brainM-PP*** | ***male*** |  |  |  |
| ***brain-AA*** | ***male*** | *This study* | ENA | PRJEB27832 |
| ***gon-AA*** | ***male*** |  |  |  |
| nd-not determined; * high probability of males; ** High probability of females | | | | |

Table S2: Statistics of assembly quality for *S. alburnoides* complex transcriptome.
